# Design Strategies for Efficient Arbovirus Surveillance

**DOI:** 10.3201/eid2304.160944

**Published:** 2017-04

**Authors:** Samuel V. Scarpino, Lauren Ancel Meyers, Michael A. Johansson

**Affiliations:** University of Vermont, Burlington, Vermont, USA (S.V. Scarpino);; Santa Fe Institute, Santa Fe, New Mexico, USA (S.V. Scarpino, L.A. Meyers);; University of Texas at Austin, Austin, Texas, USA (L.A. Meyers);; Centers for Disease Control and Prevention, San Juan, Puerto Rico, USA (M.A. Johansson);; Harvard T.H. Chan School of Public Health, Boston, Massachusetts, USA (M.A. Johansson)

**Keywords:** disease surveillance, design strategies, public health, arboviral diseases, dengue, arboviruses, viruses, Puerto Rico, vector-borne infections

## Abstract

As public health agencies struggle to track and contain emerging arbovirus threats, timely and efficient surveillance is more critical than ever. Using historical dengue data from Puerto Rico, we developed methods for streamlining and designing novel arbovirus surveillance systems with or without historical disease data.

Mosquitoborne viruses in the families *Flaviviridae* and *Togaviridae* cause substantial illness and death worldwide (*1*,*2*). Dengue is the most widespread arboviral disease, with an estimated 70–140 million cases occurring annually (*3*). Despite the large public health and economic costs of arboviruses, effective medical countermeasures are limited (*1*). Globally, primary arbovirus prevention and control efforts include personal protection, mosquito control, and clinical treatment. The success of these efforts depends on timely and accurate situational awareness: knowing spatiotemporal patterns of exposure, infection, and severity. 

Puerto Rico has an islandwide passive dengue surveillance system similar to those found in other regions with endemic dengue (*4*). Healthcare providers (clinics or hospitals) report suspected dengue cases and submit blood samples for laboratory diagnosis. This comprehensive system captures spatiotemporal variation in incidence and enables characterization of circulating viruses, but it requires substantial resources and may lack efficiency.

Here, we extend a previous approach (*5*) to designing dengue surveillance systems with 4 sets of specific public health objectives: real-time estimation of island-wide dengue cases, regional dengue cases, island-wide cases of each dengue virus serotype, and all three preceding quantities combined. Using dengue case data from 1991 through 2005, we identified a surveillance system including a subset of Puerto Rican providers that was expected to achieve these objectives efficiently and demonstrated the robustness of that system with data for 2006–2012.

## The Study

Across Puerto Rico, we analyzed the weekly number of suspect cases, laboratory-positive cases, and cases of each serotype reported during 1991–2012. For each case, we considered the patient’s municipality of residence and the identity of the reporting provider.

In designing a multipurpose dengue surveillance system, we sought to identify a small subset of providers that can provide accurate situational awareness. However, it is computationally unfeasible to evaluate all possible combinations of providers. Our procedure for solving this computational issue is described in the following sections, with a detailed description in the Technical Appendix.

Building from previous research (*6*), we design surveillance systems by sequentially adding providers that most improve system performance. To evaluate the performance of a system with respect to an objective, we repeatedly perform the following: fit multilinear models to historical reported dengue cases, use the fitted models to estimate dengue cases in another historical time period (one not used in model fitting), and quantify accuracy by using the coefficient of determination (*R*^2^) resulting from a linear regression of the estimated on the actual time series. In each repetition, we used a different combination of training data and testing data, and average all the scores across repetitions (denoted as *Ȓ*^2^). That is, we chose the set of providers that achieved the highest average out-of-sample performance (see, e.g., Technical Appendix Figure 1).

We compared our results to 3 systems in which providers were selected without historical disease data. Specifically, we selected providers on the basis of the population within 20 miles of a provider (proposed by Polgreen et al. [*7*]), the total number of patients seen (proposed by Mandl et al. [*8*]), and the diversity of the municipality of residence for patients, which does not require that each provider see an even distribution of patients; rather, providers are incorporated sequentially to achieve geographic complementarity.

**Figure 1:**
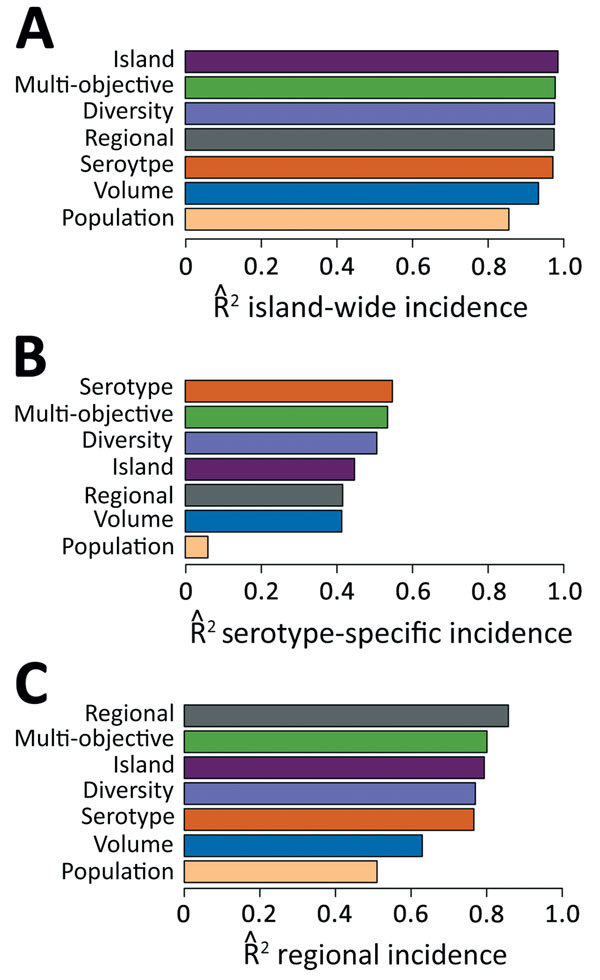
Relative surveillance system performance. The performance of the 4 optimized surveillance systems (Island, Regional, Serotype, and Multi-objective) compared with 3 alternative designs (Population, Volume, and Diversity), with respect to estimating A) island-wide cases, B) serotype-specific cases, and C) regional cases. Each system contains 22 providers. Systems are ordered from highest to lowest performance in each graph. Performance is measured by average out-of-sample across 100 different 3-year periods, resulting from linear regression of target time series (e.g., island-wide cases) on time series of cases occurring within the specified surveillance system.

We constructed surveillance systems ranging from 1 through 75 providers by using the selection algorithm for 4 objectives: island-wide cases (*Island*), island-wide cases for each of the 4 dengue virus serotypes (*Serotype*), health region-specific cases for all 8 health service regions (*Regional*), and all objectives combined (*Multi-objective*). We assessed 3 alternative systems: population coverage (*Population*), patient volume (*Volume*), and patient geographic diversity (*Diversity*). The Multi-objective system reached 99% of maximum accuracy with just 22 providers (Technical Appendix Figure 2) and performed almost as well as the systems designed specifically to achieve each objective individually (Figure 1). The Diversity system achieved 99%, 92%, and 90% of the performance of the systems specifically engineered for estimating island-wide, serotype, and regional cases, respectively, and showed similar geographic patterns to the Multi-objective system (Technical Appendix Figure 3). For individual serotypes and regions, performance was best for objectives with less sparse data (Technical Appendix Figure 4).

**Figure 2:**
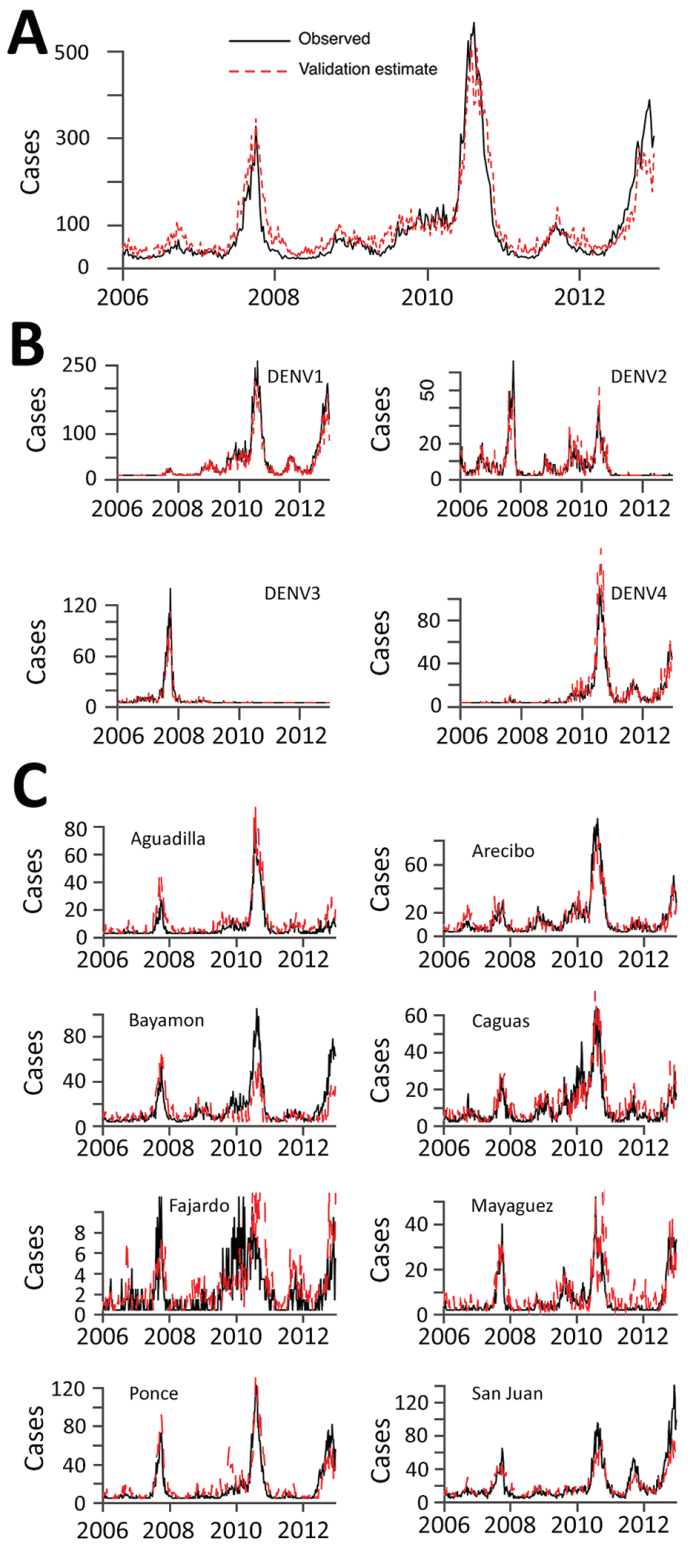
Independent evaluation of performance**.** The 22-provider Multi-objective surveillance system was designed using data before 2006 and then evaluated on data for 2006–2012 with respect to surveillance of A) island-wide, B) serotype-specific, and C) regional cases. Surveillance estimates from the 22-provider system (red) are compared with raw data from the complete passive surveillance system of 105 providers (black).

Finally, we assessed the robustness of the Multi-objective system, which offered the strongest combination of efficiency and performance. We tested it against 7 additional years’ worth of data that were withheld from the analysis. The system performed well for each of the objectives (Figure 2), achieving average values of 0.86 and 0.78 for surveillance of individual serotypes and regions, respectively, and 0.97 for surveillance of island-wide cases. Among individual serotypes and regions, all had values greater than 0.75, except for the Fajardo region, where cases were particularly sparse. 

## Conclusions

Surveillance systems are widely used to support public health efforts, but they are rarely designed systematically to achieve clear, quantifiable objectives or surveillance goals, and to do so efficiently. Articulating such public health objectives is a critical first step toward evaluating, improving, and streamlining surveillance. Here, we applied a rigorous, quantitative approach to design a dengue surveillance system that efficiently achieves several distinct public health objectives. The method flexibly and robustly maximizes information collected while minimizing the effort required. In this application, we built a multi-objective system that efficiently tracks the spatiotemporal patterns of dengue in Puerto Rico. This system is almost as informative as the systems we optimized to achieve individual objectives, and it maintained its expected performance on recent data that were withheld during the design stage.

Although surveillance goals and resources may be highly specific to the disease threat and region of concern, the proposed optimization method can be applied broadly to enhance the detection of infectious disease threats, as we have shown now for both dengue and influenza (*5*). We hypothesize that the systems we designed for dengue in Puerto Rico may also serve well for other arboviruses transmitted by *Aedes* spp. mosquitoes, given their similar transmission mechanisms and the strong out-of-sample performance of the system. In some cases, additional data (e.g., mosquito or nonhuman host surveillance) and public health goals (e.g., vector density) could be integrated into the systems. Such data were not available for this study. For newly emerging arboviruses, when historical data are not available, systems optimized for similar pathogens may provide reasonable coverage. Nonetheless, emergence dynamics may have more sporadic and explosive characteristics that may not be captured by a system designed to track spatiotemporal patterns of an endemic disease.

Public health authorities seek situational awareness at multiple geopolitical scales as well as early warning of anomalous events across a wide spectrum of biologic threats beyond arboviruses. The method we present can also be used to redesign existing surveillance systems by manually including or excluding providers during optimization. Additionally, the method is well suited to integrating diverse data streams, such as climatic, mosquito vector, pharmacy, or digital data (*9*).

In an era of “right-sizing,” quantitative development and evaluation are critical to the design, redesign, justification, and benchmarking of surveillance efforts. Given limited public health budgets on all scales, methods such as the one we present are critical to the future reliability and sustainability of infectious disease surveillance.

Technical Appendix Discussion of the methods and algorithm for provider selection with the goal of surveillance system optimization.
