# Supplementary material for: Design Strategies for Efficient Arbovirus Surveillance
Source: Emerg Infect Dis. 2017 Apr;23(4):642–4. doi: 10.3201/eid2304.160944 (PMC5367434; doi:10.3201/eid2304.160944)
Supplement: Technical Appendix — Discussion of the methods and algorithm for provider selection with the goal of surveillance system optimization. [file 16-0944-Techapp-s1.pdf]

Article DOI: http://dx.doi.org/10.3201/eid2304.160944

Design Strategies for Efficient Arbovirus Surveillance

Methods

Simulating Provider Data, 1991–1998

The identities of the submitting providers were not included in dengue reports before 1999. However, these identities are critical to our optimization methods. Thus, we used a simulation method to assign each pre-1999 case to a specific provider, based on post-1999 data linking providers to patient municipalities. Each pre-1999 report was either assigned to a known provider or designated as unknown, as follows:

1) Estimating the fraction of cases with known and unknown providers for each municipality. During 1999–2005, a weekly average of 10% of the reported suspected cases did not identify a provider (range 0%–37%), with the proportion varying by municipality and increasing at times of high case volumes. For each municipality, we stratified the 1999–2005 reports into 5 equal-width bins based on the observed island-wide cases when each case was reported. Then, for each municipality (m)-cases bin (b) combination, we calculated the proportion of cases with a known provider ($k_{m,b}$).

2) Estimating distribution of cases across providers, for each municipality. Using 1999–2005 case reports with known providers, we calculated the fractions of cases in each of the 78 municipalities (m) that were reported by each of the 105 providers (p)($a_{m,p}$).

3) Assigning pre-1999 cases to providers. For the 1991–1998 data, which contain the patient's municipality of residence but not the reporting provider, we simulated provider identities by multiplying the quantities described in the first two steps. That is, the number of cases from a given municipality (m) assigned to a given provider (p) for a given time period depended on the island-wide cases at the time (b), and was set equal to the product of the total number of dengue cases reported in m during that period, the estimated fraction of cases from that municipality with a known provider ($k_{m,b}$), and the estimated fraction of dengue cases from m seeking care from p ($a_{m,p}$). Last, fractional cases were rounded to integers.

Surveillance Objectives

For each of the surveillance objectives, we formulated specific quantities to be estimated from the surveillance data: for island-wide cases, the total number of laboratory-positive cases by week; for serotype cases, the total number of cases of each of the 4 serotypes reported by week; and for regional cases, the number of confirmed cases in each of the 8 health service regions by week.

In designing a multipurpose dengue surveillance system, we sought to identify a relatively small subset of providers that could provide accurate real-time estimates of these quantities. However, it is computationally unfeasible to evaluate all possible combinations of providers. For example, an exhaustive analysis of all subsets of 75 providers from the full set of 105 providers would require 1.6×10^{26} evaluations. Rather than performing an exhaustive search, we used a more efficient procedure for identifying providers for inclusion in the surveillance system, as described in the following sections.

Surveillance System Optimization

We designed surveillance systems using a greedy algorithm that sequentially adds providers that most improve the performance of the system. Starting with the set of all possible providers P (in this case, all clinics in Puerto Rico historically reporting dengue cases), we selected a set of providers, S, which initially has no members. At each step, we added the provider that is expected to yield the highest value of our objective function, f.

The Objective Function

To evaluate the performance of a given system (set of providers S) with respect to the surveillance objectives listed previously, we repeatedly performed the following three-step procedure: 1) fit multiple linear models relating historical data from the surveillance system in question to actual dengue cases, 2) use the fitted models to estimate dengue cases in another historical time period (that was not included in the model fitting procedure), and 3) quantify the accuracy of those estimates. In each repetition, we used a different combination of training data and testing data, and ultimately combined all the accuracy estimates (across all objectives and repetitions) into a single objective function.

Our overarching surveillance objective was a set, G, of up to 13 different subobjectives, g (estimating dengue cases across the whole island, in each of the 8 geographic regions, and for each of the 4 different serotypes). When evaluating a subset of providers, S, we fit multiple linear models (one per subobjective) given by

$$S\theta(g, S): Y_{g,t} = a_g + \sum b_{s,g} X_{s,g,t} + \varepsilon_t,\text{s}$$

to the testing data, where $Y_{g,t}$ are the actual cases with respect to objective g at time t (for example, island-wide dengue virus serotype 1 (DENV-1) cases in a particular week), $X_{s,g,t}$ are the cases with respect to objective g reported by provider s at time t, a_g and $b_{s,g,}$ are the model coefficients, and ε_t is a zero-mean normally distributed error term.

After estimating the coefficients of each subobjective model by using the training data, we used the models to predict the case quantities during the testing period and quantify the accuracy of the predictions by calculating R^2 values. Ultimately, we aggregated the accuracy measurements into the single objective function given by $f\,(S, G, D)$.

$$\sum R^2(\theta(g, S), d) w_g w_d,\ \text{g}\in\text{G d}\in\text{D}$$

where $R^2(\theta(g, S), d)$ represents the out-of-sample performance of system S on objective g with testing-training data combination d; D represents the set of testing-training data combinations used in the evaluation; and w_g and w_d are weights indicating the contribution of each objective and dataset to the objective function, where each sum to 1, $\sum g \in G\ w_g = 1$ and $\sum d \in D\ w_d = 1$. For simplicity we refer to $R\ (\theta(g, S), d)$ as R'.

We built surveillance networks for 4 different objective functions (each consisting of a distinct combination of subobjectives): 1) overall island-wide cases (*Island*), 2) island-wide cases for each of the 4 DENV serotypes, with each serotype given a 1/4 weight (*Serotype*), 3) regional dengue cases for each of the 8 health service regions, with each region given a 1/8 weight (*Regional*), and 4) the 3 prior objectives weighted equally (*Multi-objective*), resulting in weights of 1/12 for each serotype case, 1/32 for each regional case, and 1/3 for each island-wide case.

Provider Selection Algorithm

At each step in the optimization, we considered all providers that had not yet been included in the system, and selected the one that produced the maximum value of f. Let S_n denote the surveillance system at step n in the optimization. The iterative selection proceeds as follows:

1) For each provider $x \in P | x \notin S_n$, create a candidate system $S_{n,x} = \{S_n, x\}$ and calculate $f(S_{n,x}, G, D)$.

2) Identify the provider x that maximizes the expected improvement in performance $f(S_{n,x}, G, D) - f(S_n, G, D)$.

3) Add x to S.

4) Repeat.

***Volume*-based design:** We selected providers sequentially based on the total number of patients seen during 1990–2005.

***Diversity*-based design:** We used a greedy algorithm to maximize the Shannon diversity index, H, of the municipality of residence for patients captured by the surveillance system S. If there are M municipalities and the proportion of patients in S from municipality i is p_i, then the Shannon diversity for S is:

$$H_S = -\sum p_i \ln p_i$$

This quantity is maximized when $p_i = 1/M$, that is, when the municipalities contribute equal numbers of patients to S. This does not require that each provider see a uniform distribution of patients, and providers are incorporated sequentially to achieve geographic complementarity. This procedure resembles the *Population*-based model, but maximizes the diversity of patient residences rather than the number of patients.

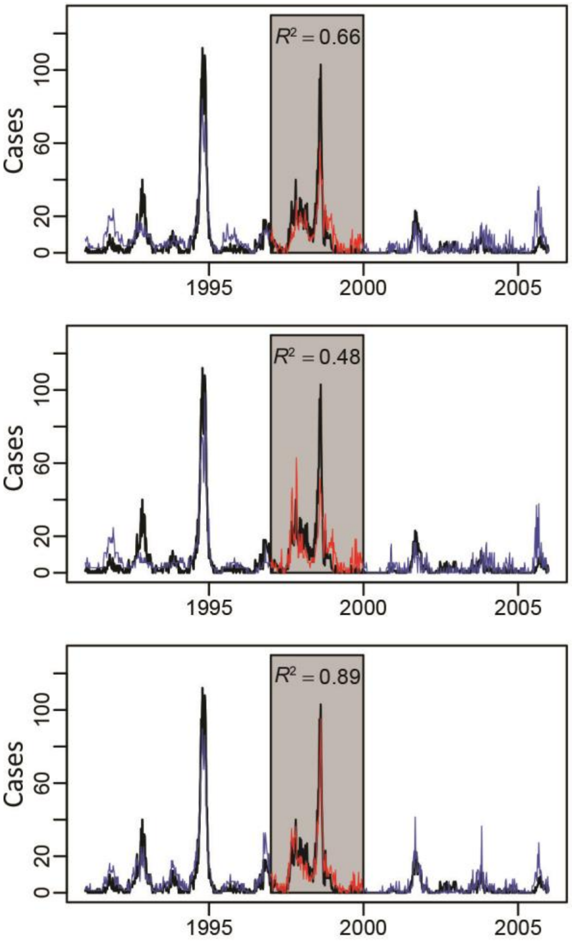

Technical Appendix Figure 1. Provider selection for dengue surveillance in the Mayaguez health service region. To design a system for regional-level dengue surveillance, we evaluated the performance of different combinations of providers across each of the 8 health service regions. This figure illustrates a single step in the selection process for one of the health service regions after 1 provider has already been included. Dengue incidence in the Mayaguez region is shown in black for the period of 1991–2005, and serves as the response variable in the regression-based provider selection method. The panels show 3 candidate providers under evaluation for subsequent inclusion as the second provider selected for the system. We combined data from each candidate with data from the first provider already incorporated in the system. We then performed linear regression of total Mayaguez incidence on the combined data during the 1991–1996 and 2000–2005 time periods (blue), and made out-of-sample predictions (red). Performance is quantified by the out-of-sample R^2. This process is repeated 100 times with random 3-year intervals withheld for out-of-sample evaluation. The candidate provider delivering the highest average R^2 across the 100 trials is selected for inclusion. In this example, the provider associated with the bottom panel is more informative than the alternatives.

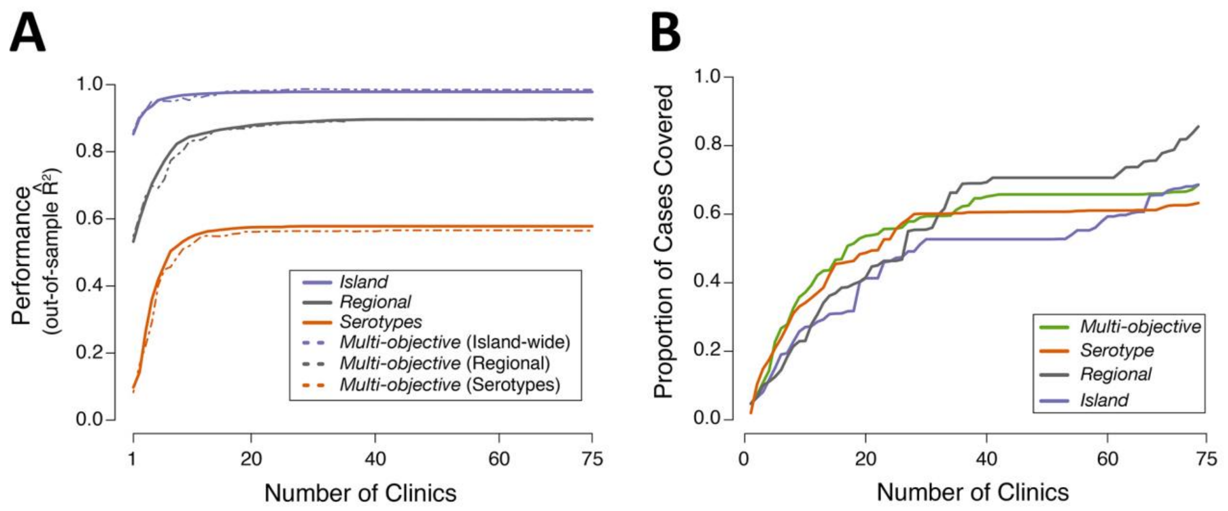

Technical Appendix Figure 2. Performance curves for optimized dengue surveillance systems. As providers are added to the systems, A) estimation of island-wide, regional, and serotype cases improves and then levels, as quantified by average out-of-sample R^2, whereas B) the proportion of DENV cases occurring at providers within the system increases more gradually. Some providers were estimated to have reported either zero or very nearly zero cases during the study period, which is why the proportion of cases covered does not increase with each additional provider. The maximum performance remains less than 1.

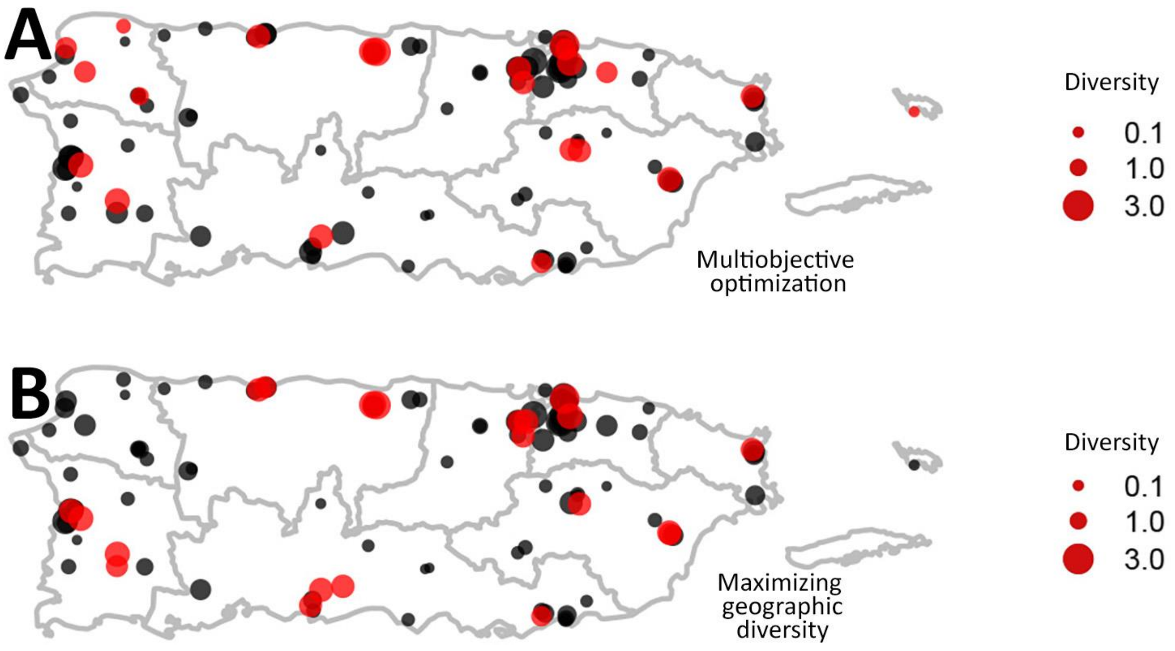

Technical Appendix Figure 3. Location and patient geographic diversity of selected providers. The 22 providers selected A) under multi-objective optimization (*Multi-Objective*) and B) when maximizing the geographic diversity of patients (*Diversity*) are indicated in red and the remaining 92 providers in black. Circle size reflects the Shannon diversity of patient municipalities of a given provider. The lines indicate

the boundaries of the Puerto Rico health regions. The islands of Culebra and Vieques (right) are part of the Fajardo health region (northeastern corner).

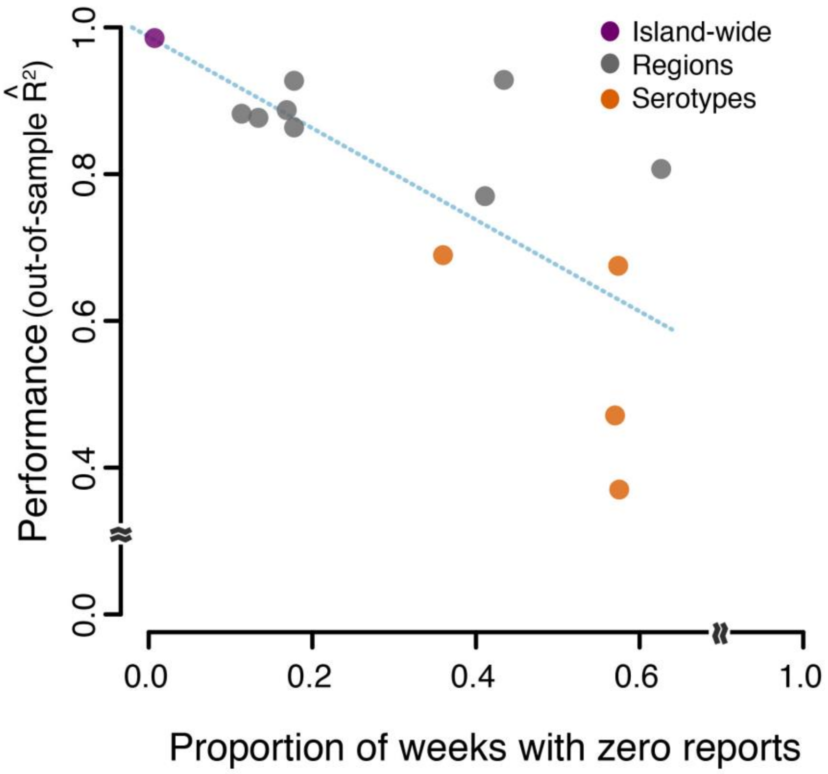

Technical Appendix Figure 4. Performance decreases as sparsity of training data increases. For each of the 13 different surveillance subobjectives (island-wide incidence, incidence in each of the 8 health service regions, and incidence for each of the 4 serotypes), we plot the average out-of-sample R^2 for the best combination of 22 providers against the proportion of weeks with zero reported cases in the training period time series data (1991–2005). For example, for dengue virus serotype 1 (DENV-1), we find the combination of 22 providers that maximizes performance, and plot the resulting performance against the proportion of weeks during 1991–2005 without a reported case of laboratory-confirmed DENV-1. Performance is measured by average out-of-sample R^2 across 100 different 3-year periods, resulting from linear regression of a target time series (e.g., all DENV-1 cases) on the time series of cases occurring within the candidate set of providers. The least-squares regression line relating performance to data quality is plotted (blue dashes).
